# Inhibition of Aurora Kinase B Is Important for Biologic Activity of the Dual Inhibitors of BCR-ABL and Aurora Kinases R763/AS703569 and PHA-739358 in BCR-ABL Transformed Cells

**DOI:** 10.1371/journal.pone.0112318

**Published:** 2014-11-26

**Authors:** Anna L. Illert, Anna K. Seitz, Christoph Rummelt, Stefanie Kreutmair, Richard A. Engh, Samantha Goodstal, Christian Peschel, Justus Duyster, Nikolas von Bubnoff

**Affiliations:** 1 Clinic for Internal Medicine 1, Hematology, Oncology and Stem Cell Transplantation, Freiburg University Medical Center, 79106 Freiburg, Germany; 2 Department of Internal Medicine III, Technical University of Munich, 81675 Munich, Germany; 3 The Norwegian Structural Biology Centre, Departments of Chemistry and Pharmacy, University of Tromsø, Tromsø, Norway; 4 EMD-Serono Research and Development Institute, Inc., Billerica, Massachusetts, United States of America; Institut de Génétique et Développement de Rennes, France

## Abstract

ABL tyrosine kinase inhibitors (TKI) like Imatinib, Dasatinib and Nilotinib are the gold standard in conventional treatment of CML. However, the emergence of resistance remains a major problem. Alternative therapeutic strategies of ABL TKI-resistant CML are urgently needed. We asked whether dual inhibition of BCR-ABL and Aurora kinases A-C could overcome resistance mediated by ABL kinase mutations. We therefore tested the dual ABL and Aurora kinase inhibitors PHA-739358 and R763/AS703569 in Ba/F3- cells ectopically expressing wild type (wt) or TKI-resistant BCR-ABL mutants. We show that both compounds exhibited strong anti-proliferative and pro-apoptotic activity in ABL TKI resistant cell lines including cells expressing the strongly resistant T315I mutation. Cell cycle analysis indicated polyploidisation, a consequence of continued cell cycle progression in the absence of cell division by Aurora kinase inhibition. Experiments using drug resistant variants of Aurora B indicated that PHA-739358 acts on both, BCR-ABL and Aurora Kinase B, whereas Aurora kinase B inhibition might be sufficient for the anti-proliferative activity observed with R763/AS703569. Taken together, our data demonstrate that dual ABL and Aurora kinase inhibition might be used to overcome ABL TKI resistant CML.

## Introduction

Chronic myeloid leukemia (CML) is a neoplastic disease of hematopoietic stem cells triggered by the oncogene BCR-ABL. This fusion gene is the result of a reciprocal translocation between chromosomes 9 and 22 and characterized by constitutively activation of the BCR-ABL tyrosine kinase [1]–[3]. Since 2002, the treatment of CML was revolutionized by the introduction of the ATP-competitive inhibitor imatinib mesylate (IM, Gleevec), a BCR-ABL tyrosine kinase inhibitor (TKI) with strong activity against the tyrosine kinases PDGFR, cKit and Abl. [4]–[7]. The clinical use of Imatinib resulted in a significantly improved prognosis, response rate, overall survival, and patient outcome in CML patients compared to previous therapeutic regimens [8]–[10] and made it the gold standard in conventional treatment of CML [11]. However, some CML patients in chronic phase and a substantial proportion in accelerated phase and blast crisis are either initially refractory to IM or loose IM sensitivity over time and experience relapse [12]–[18]. Several mechanisms leading to IM resistance have been characterized during the last years: most commonly, mutations in the BCR/ABL domain confer IM resistance, either by altering IM binding characteristics or through indirect modulation of kinase function, which are often associated with secondary (acquired) resistance [19]. In this sense, kinase domain mutations are the most frequently identified mechanism associated with relapse [20]–[26]. Substitution of threonine with isoleucine at residue 315 (T315I gatekeeper mutation) is the most prevalent mutation (14%) in IM- resistant patient [27] followed by the p-Loop Mutation Y253F/H [17], [18]. Second-generation BCR-ABL TKIs nilotinib (Tasigna) and dasatinib (Sprycel) showed significant activity in clinical trials in patients resistant to imatinib therapy [28]–[35], except in those with the T315I BCR-ABL gatekeeper mutation [20], [26], [36], [37]. However, the prognosis of Imatinib refractory or intolerant chronic myelogenous leukemia and advanced Ph^+^ acute lymphoblastic leukemia is still poor and new therapies are urgently needed for those patients. Aurora kinase inhibitors (AKI) have recently emerged as promising drugs in CML therapy, but it has not been entirely clear whether the AKI apoptotic effect is due to BCR-ABL or Aurora kinase (A or B) inhibition and whether dual inhibition of BCR-ABL and Aurora kinases could overcome resistance mediated by ABL kinase mutations. Members of the Aurora kinase family represent a new and promising target for anticancer therapeutics. Within this family, Aurora kinases are highly homologous and conserved serine-threonine protein kinases that play a key role in mitosis [38]–[42]. In mammalian cells Aurora kinases are comprised of three family members: Aurora kinases A, B and C. Aurora kinase A activity and protein expression increases from late G_2_-phase through Mitosis and is required for centrosome-maturation and -separation, mitotic entry, and spindle assembly [43]. Selective Aurora A inhibition due to inhibition of Thr288 autoposphorylation leads to p53-dephosphorylation, monopolar spindel formation with consecutive G_2_/M arrest and apoptosis [44]–[47]. In contrast, Aurora kinase B is the catalytic part of the chromosomal passenger complex (CPC) and critical not only for chromosomal condensation, segregation and bi-orientation but also for the spindle-assembly checkpoint and final stages of cytokinesis [48]–[50]. Classically, selective Aurora B inhibition leads to polyploidy and apoptosis [51]–[53] by inhibition of Histone-3 phosphorylation at serine 10, a well-known down-stream-target of Aurora B. Expression of Aurora C seems to be limited to the testis and its role has not been well defined yet. Both, Aurora kinases A and B, have been linked to tumorigenesis with the frequent finding of gene amplification and/or overexpression in several malignancies [54]–[59] including CML, where it was shown that BCR-ABL regulates Aurora A [60] and B inhibition (Figure S1). Furthermore, a functional cross-talk between Aurora A and the p53- and p73-dependent apoptotic pathway in cancer cells was reported [61].

The oncogenic role of Aurora kinases as well as their crucial role in cell cycle division makes them an attractive potential target in anti-cancer therapy. A growing number of Aurora kinase inhibitors have been developed during the past years and entered successfully clinical phase I or II studies like MK-5108, MLN8054, MLN8237, PHA-739358, AZD1152, AT92830, MSC1992371A, PF-03814735 and R763/AS703569 [44], [45], [62]–[83].

Here we asked, whether dual inhibition of BCR-ABL and Aurora kinases could overcome resistance mediated by ABL kinase mutations and therefore tested the dual ABL and Aurora kinase inhibitors PHA-739358 and R763/AS703569 in BaF3- cells expressing wild type (wt) or TKI-resistant BCR-ABL mutants. We show that both compounds exhibited strong anti-proliferative and pro-apoptotic activity in ABL TKI resistant cell lines including the T315I mutation. Furthermore, we were able to identify a drug resistant Aurora B mutant that renders cells partially resistant to PHA-739358 and R763/AS703569 in vitro with a cell-based screen of resistance. With the help of this mutant, we could show, that Aurora B is an important target with great potential in further anti-cancer drug development.

## Materials and Methods

### Inhibitors

PHA-739358 was kindly provided by Nerviano Medical Sciences, Milan, Italy. It was dissolved at 20 mM in dimethyl sulfoxide (DMSO) and stored at −20°C. Kinase inhibition profile and IC50s of PHA-739358 (Danusertib) are published by Fancelli et al. [84], [85] R763/AS703569 was a kind gift from EMD-Serono (Rockland, MA). A stock solution of R763/AS703569 (10 mmol/L; in DMSO) was stored at −20°C. Kinase inhibition profile and IC50s of R763/AS703569 are published by McLaughlin [86].

### Cell culture, transfection and cell cycle synchronization

Parental Ba/F3 cells were obtained from DSMZ (Braunschweig, Germany). Ba/F3 cells were cultured under standard conditions in RPMI 1640 growth media (Gibco, Karlsruhe, Germany) containing 10% fetal bovine serum, 200 U penicilin per mL, and 200 g streptomycin per mL (Gibco, Karlsruhe, Germany). IL3-dependend Ba/F3 cells were supplemented with 2 ng/mL interleukin-3 (IL-3; R&D, Wiesbaden, Germany). Ba/F3 p185wt cells were infected with retrovirus (pBabe-puro, pBabe-AurA, pBabe-AurB) and puromycin-treated for selection. For synchronisation in G_0/1_, cells were serum starved for 72 h in the presence of 0,5% FCS.

### DNA constructs

BCR-ABL p185 was cloned as described previously [87]. Human Aurora A and B wt cDNAs in pEGFP-C1 were obtained from E. Nigg (Martinsried, Germany). Mutant forms of Aurora A and B were engineered in pEGFP-C1AurA/AurB using the QuickChange Mutagenesis kit (Stratagene, Amsterdam, Netherlands), subcloned in pCR-Blunt II-Topo or pCR-2.1-TOPO (Invitrogen, Karlsruhe, Germany) and subsequently into pBabe-puro (Addagene, Cambridge, MA, USA). All constructs were verified using automated sequencing.

### Proliferation

Cell viability was assessed using a MTS (3-(4,5-dimethylthiazol-2-yl)-5-(3-carboxymethoxyphenyl)-2-(4-sulphophenyl)-2H-tetrazoliumsalz)-based method (CellTiter; Promega, Madison, WI). Briefly, 10^5^ cells were seeded in triplicates in 96-well flat-bottom microtiter plates, treated as indicated and absorption measured at 490 nM. The cellular IC_50_ value corresponds to the concentration of 50% growth inhibition. IC_50_ values were calculated by graphic exploration of the dose-effect curve.

### Cell cycle and apoptosis

For dynamic cell cycle analysis (BrdU-incorporation) cells were pulsed with 10 μM BrdU for 1 h and stained with anti-BrdU according to a standard protocol (BD Biosciences). For static cell cycle analysis, cells were stained with propidium iodide (25 mg/ml PI; 100 µg/ml RNAse in PBS; Sigma-Aldrich, Heidelberg, Germany). Cell cycle distribution and apoptosis was determined by PI or two-dimensional (fluorescein and PI) flow cytometry. Analysis of DNA content occurred in the linear mode, apoptosis measuring in the logarithmic mode of the FL2 channel. Results were quantified using FlowJo software (Tree Star Inc., Ashland, OR). Cells with DNA content <2n were considered apoptotic (sub-G1).

### Western blot

Cells were cultured for 2.5 h without (w.o.) or in the presence of inhibitor at the indicated concentrations. After cell lysis, sonification, and measuring of the protein concentration using *BioRad Protein Assay* (Biorad, Munich, Germany), protein extracts were electrophoretically separated on SDS-PAGE gels and immunoblotted with indicated antibodies. Anti-Aurora A, anti-Aurora B, Anti- pHistone-3 (Serine 10), and anti-β-Actin antibodies were obtained from Sigma-Aldrich (Heidelberg, Germany), Anti-Flag (M2) antibody from Stratagene (Heidelberg, Germany). Anti-Abl antibody (8E9) was purchased from BD Biosciences (San Jose, CA), anti-STAT5 antibody (C-17) from Santa Cruz Biotechnology Inc. (Heidelberg, Germany). Detection of phosphotyrosine was performed using a mixture of the antibodys 4G10 (Millipore, Schwalbach, Germany) and PY20 (BD Biosciences, San Jose, CA). pSTAT5 antibody was a kind gift from T. Wheeler (Hamilton, New Zealand; Wheeler et al., 2001). Bands were visualized using SuperSignal Chemoluminescence substrate (Pierce, Rockford, USA).

### Structural modeling

Graphical inspection and structural superposition was done with PyMOL (DeLano W.L., The PyMOL Molecular Graphics System (2002), http://www.pymol.org). Individual features of inhibitor interactions and geometries were examined also using the electron density server and 3D-Ligand Interaction analyzer, as linked in the PDB server pages.

## Results

### PHA-739358 and R763/AS703569 exert anti-proliferative effects in BCR-ABL negative and positive Ba/F3 cells

**Figure 1:**
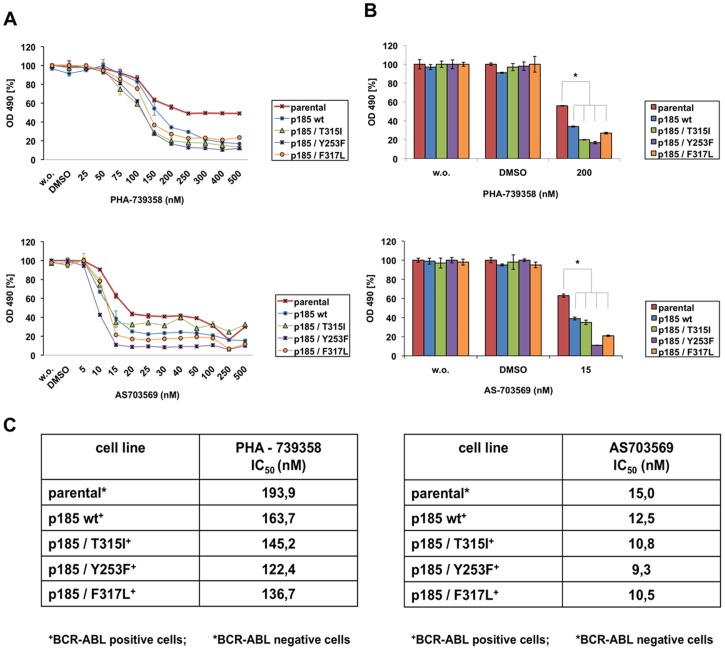
Aurora Kinase inhibitors PHA-739358 and R763/AS703569 compromise cell proliferation and viability at different concentrations in BCR-ABL negative and positive Ba/F3 cells independent of the BCR-ABL mutation status. (A) Dose-effect curves for treatment with the indicated concentrations of PHA-739358 and R763/AS703569 in Ba/F3 and Ba/F3 p185 cells, including the IM-resistant mutants T315I, Y253F and F317L, after 48 h. Effects on cell proliferation were assessed by MTT assay. Shown is one representative experiment of three experiments performed. The percentage of cell growth was normalized to the growth of untreated control cells. (B) Bar graph quantifying cell proliferation after exposure to 200 nM PHA-739358 or 15 nM R763/AS703569 for 48 h. Untreated and DMSO-treated cells were used as controls. Shown is one representative experiment of three experiments performed. Values are expressed as mean of triplicates ± SD. The difference between Ba/F3 p185 wt and BCR-ABL mutants is statistically significant. (C) IC_50_ values of BCR-ABL positive and negative Ba/F3 cells, calculated from the results after 48 h of incubation with PHA-739358 or R763/AS703569.

To determine active concentrations of both inhibitors, we treated BCR-ABL negative and positive Ba/F3 cells, including the IM-resistant BCR-ABL mutants T315I, Y253F and F317L, with increasing concentrations of PHA-739358 and R763/AS703569. PHA-739358 strongly inhibited cell proliferation in parental and BCR-ABL expressing Ba/F3 cells in a dose-dependent manner. BCR-ABL mutational status did not affect the anti-proliferative response to PHA-739358 or R763/AS703569 (Figure 1A). Thus, sensitivity of individual BCR-ABL mutants to PHA-739358 and R763/AS703569 did not correlate with the degree of resistance to IM, and the highly imatinib resistant BCR-ABL/T315I mutation displayed similar dose-response compared to BCR-ABL wt cells. Accordingly, IC_50_ values were in a range of 150 nM for PHA-739358 and 10 nM for R763/AS703569, independent of the BCR-ABL mutation status (Figure 1A). Thus, both inhibitors exerted anti-proliferative effects in a dose-dependent manner and independent of the BCR-ABL mutation status, with R763/AS703569 displaying significantly lower IC_50_ values (Figure 1B).

### PHA-739358 and R763/AS703569 effectively inhibit BCR-ABL and Aurora Kinases

**Figure 2:**
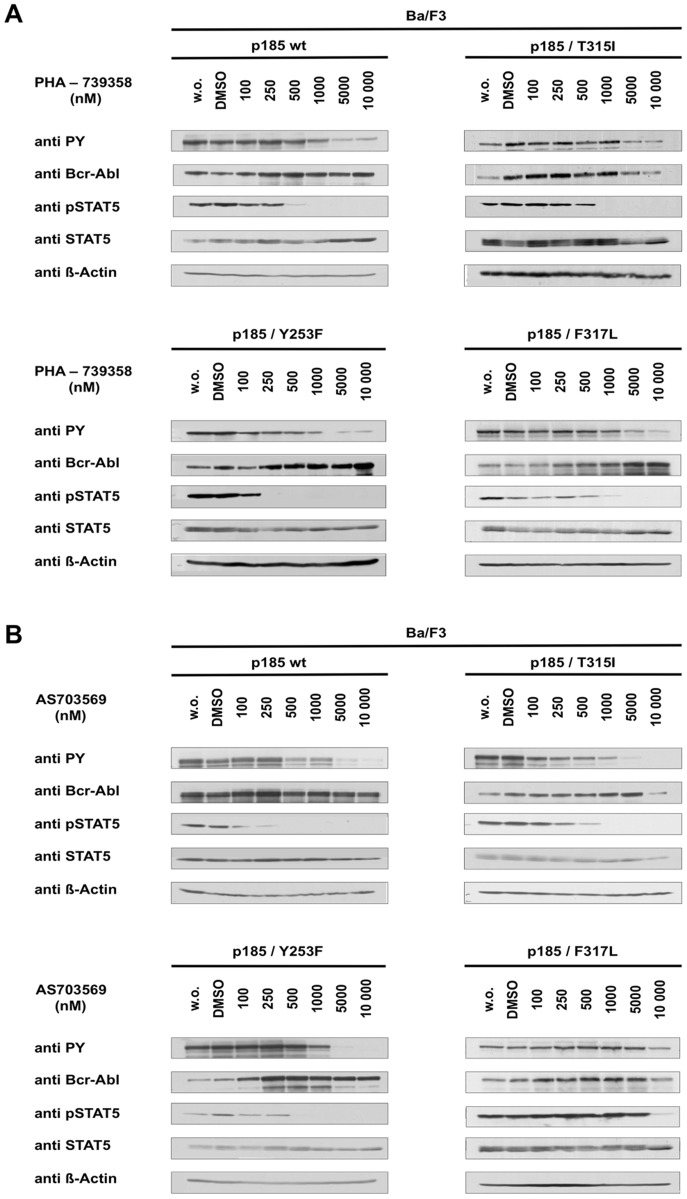
PHA-739358 and R763/AS703569 reduce BCR-ABL kinase activity at comparable concentrations and independent of the BCR-ABL mutation status. Ba/F3 p185 wt and IM-resistant T315I, Y253F, and F317L mutants cells were exposed to increasing concentrations of PHA-739358 (A) or R763/AS703569 (B) for 2.5 h and assessed for phosphorylation status of BCR-ABL and its downstream target STAT5 by western blot analysis. Untreated and DMSO treated cells were used as a controls.

It has been described that AKIs can influence BCR-ABL kinase activity by inhibiting BCR-ABL autophosphorylation and phosphorylation of its downstream target STAT5. Therefore we asked whether the anti-proliferative effect of PHA-739358 and R763/AS703569 is a result of BCR-ABL or Aurora kinase inhibition. Treatment with PHA-739358 (Figure 2A) or R763/AS703569 (Figure 2B) resulted in strong inhibition of BCR-ABL kinase activity in BCR-ABL positive Ba/F3 cells. Effective concentrations for inhibition of BCR-ABL kinase activity and signaling were around 1000 nM and above for PHA-739358 and in the range of 500 nM for R763/AS703569. Inhibition of BCR-ABL phosphorylation occurred at similar concentrations for Ba/F3 p185wt, p185/T315I and p185/Y53F cells. Surprisingly, higher concentrations of R763/AS703569 but not of PHA-739358 were required to inhibit BCR-ABL phosphorylation in cells expressing BCR-ABL/F317L. Thus, for PHA-739358 cellular IC_50_ values were in the range of the concentrations where inhibition of BCR-ABL occurred, whereas -in case of R763/AS703569- IC_50_ values were more than 50-times lower than the concentrations needed for BCR-ABL kinase inhibition. Taken together, these data provide evidence, that BCR-ABL kinase inhibition might be, at least in part, responsible for the anti-proliferative effect of PHA-739358, but not for R763/AS703569.

**Figure 3:**
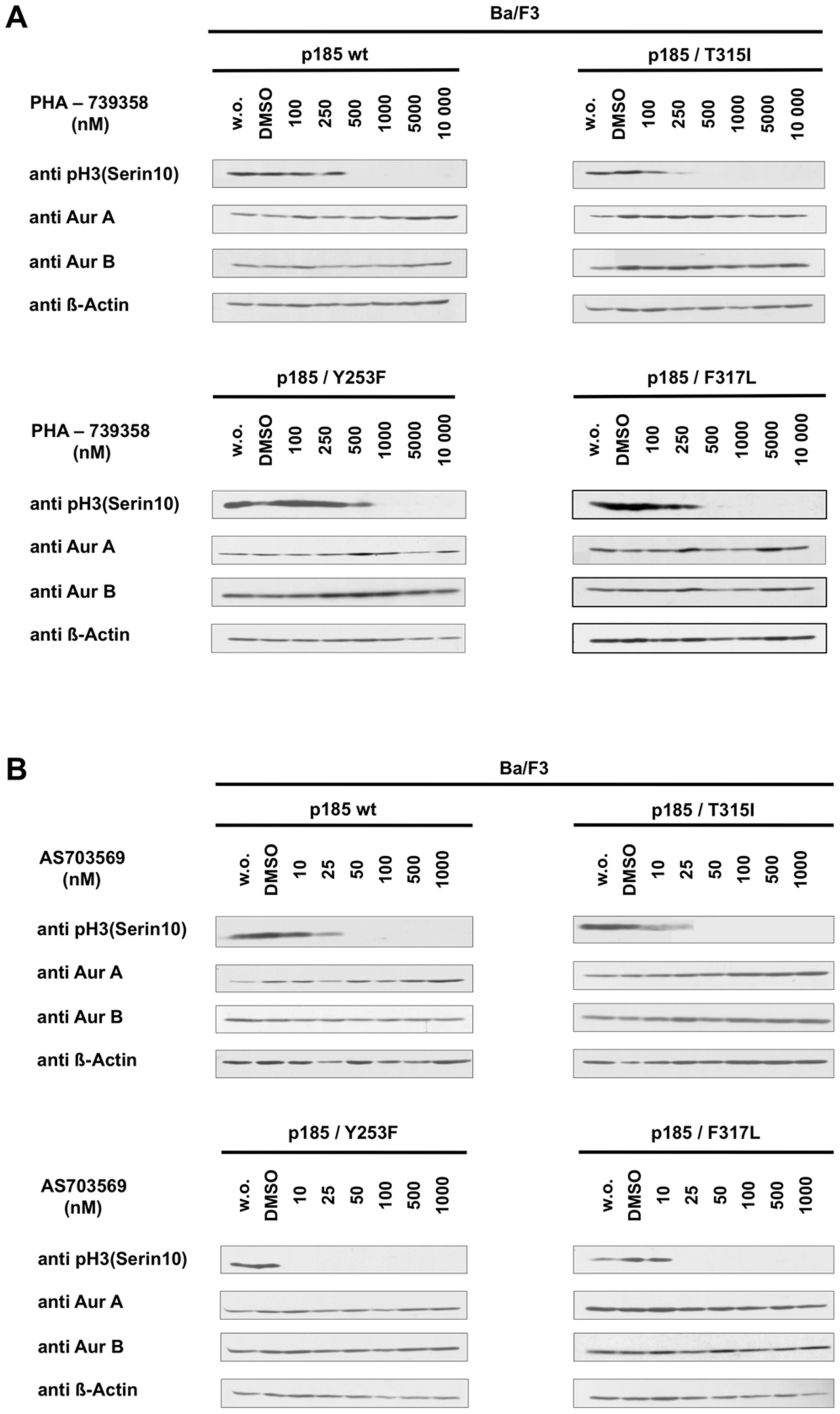
Inhibition of Aurora kinase activity occurs at different concentrations of PHA-739358 and R763/AS703569. Ba/F3 p185 wt, p185/T315I, p185/Y253F and p185/F317L cells were treated with indicated concentrations of PHA-739358 (A) or R763/AS703569 (B) for 2.5 h. Aurora Kinase activity assessed by phosphorylation status of Histone-H3 at Serine-10 was analyzed using immunoblotting. Control cells were left untreated or exposed to DMSO.

To assess inhibitory activity of PHA-739358 and R763/AS703569 on Aurora kinases, phosphorylation of Histone-3 (H3) on Serine-10, a bone-fide Aurora B target [50], [52], [85], [88], was determined. BCR-ABL positive cells exposed to PHA-739358 (Figure 3A) or R763/AS703569 (Figure 3B) showed a distinct reduction of phosphorylation on H3-Ser10, however at different concentrations. While with PHA-739358 Aurora kinase inhibition was observed at 500 nM, 20 times lower concentrations of R763/AS703569 were sufficient to obtain the same effect on H3-ser10 phosphorylation. Thus, low concentrations of R763/AS703569 needed to suppress Histone-3 phosphorylation corresponded to the cellular IC_50_ value. Taken together, anti-proliferative effects of PHA-739358 can be likewise attributed to BCR-ABL and Aurora kinase inhibition, whereas the anti-proliferative activity seen with R763/AS703569 is exclusively due to Aurora kinase inhibition.

### PHA-739358 and R763/AS703569 induce cell division failures and apoptosis in BCR-ABL negative and positive Ba/F3 cells

**Figure 4:**
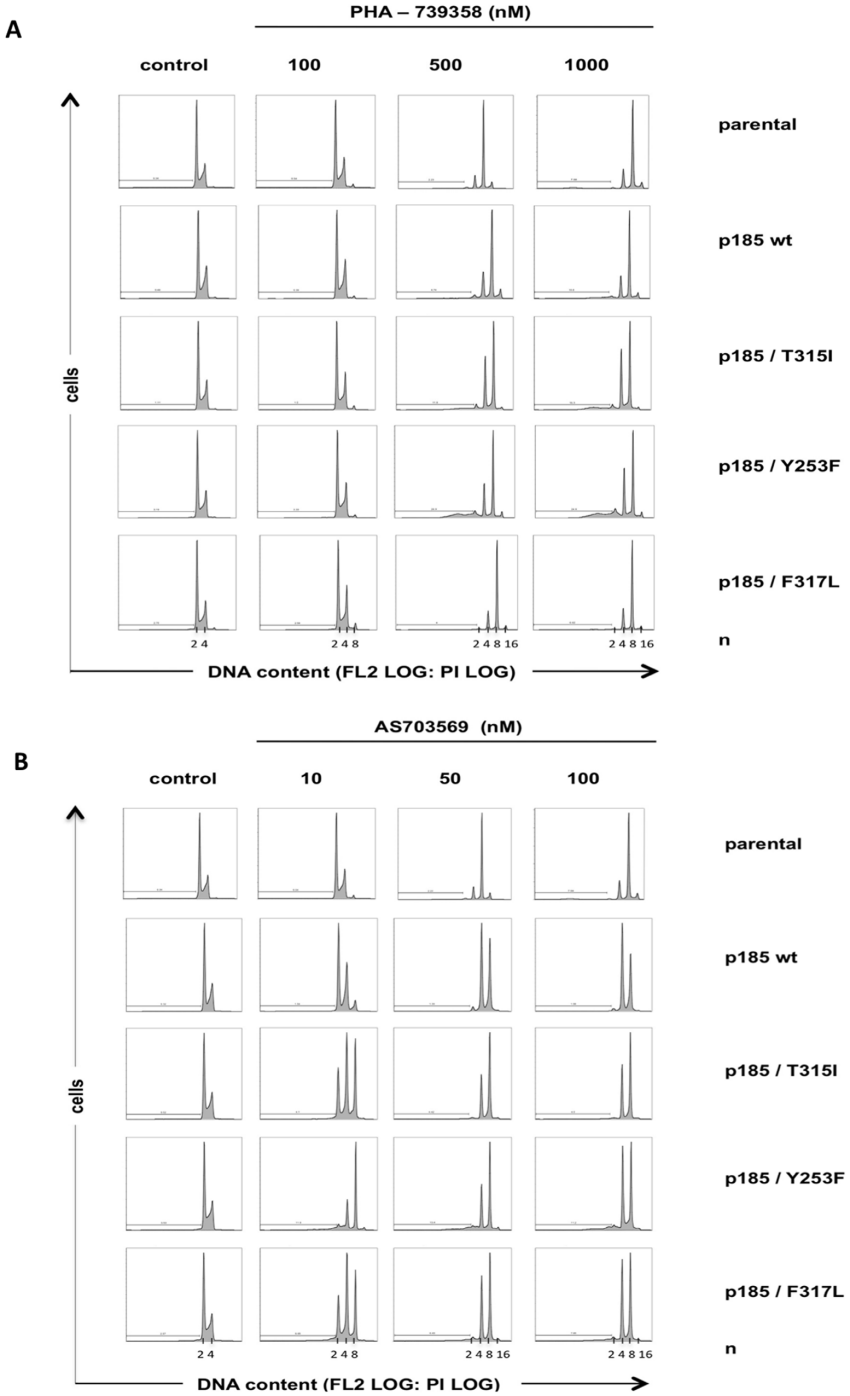
Suppression of Aurora B kinase activity by PHA-739358 or R763/AS703569 inhibits cell division and induces apoptosis in BCR-ABL negative and positive Ba/F3 cells. Ba/F3 and Ba/F3 p185 cells comprising the T315I, Y253F, and F317L mutations were exposed to the indicated concentration of PHA-739358 (A) or R763/AS703569 (B). After 24 h analysis of DNA content and apoptotic fraction of PI-stained cells were assessed by flow cytometry. Untreated cells served as control. Apoptosis was measured as the percentage of cells of sub-G1 DNA content in the FL2 channel in a logarithmic scale.

Aurora Kinases A and B play key roles in regulation of mitotic processes [38], [40]. Inhibition of these kinases leads to mitotic defects and prevents cytokinesis [39], [89]. In order to determine which Aurora kinase is the relevant target for the used inhibitors, static cell cycle analyses were performed by flow cytometry. Exposure to PHA-739358 at 500 nM or above induced endoreduplication and generated polyploid cells with 8n and up to 16n DNA-content with subsequent apoptosis (Figure 4A). We observed this effect not only in BCR-ABL positive cells but also in parental, BCR-ABL negative cells. As expected, treatment with lower concentrations of PHA-739358 did not change cell cycle profiles because of insufficient Aurora kinase inhibition. R763/AS703569 abolished cell division in BCR-ABL negative and positive Ba/F3 cells, resulting in accumulation of cells with DNA content ≥4n (Figure 4B). Polyploidisation already became apparent at very low concentrations (10 nM R763/AS703569) and increased at higher concentrations. Interestingly, a significant sub-G1 DNA content became apparent with PHA-739358 at concentrations were inhibition of both Aurora as well as BCR-ABL kinase was observed.

### Identification of drug resistant Aurora kinase mutants

**Figure 5:**
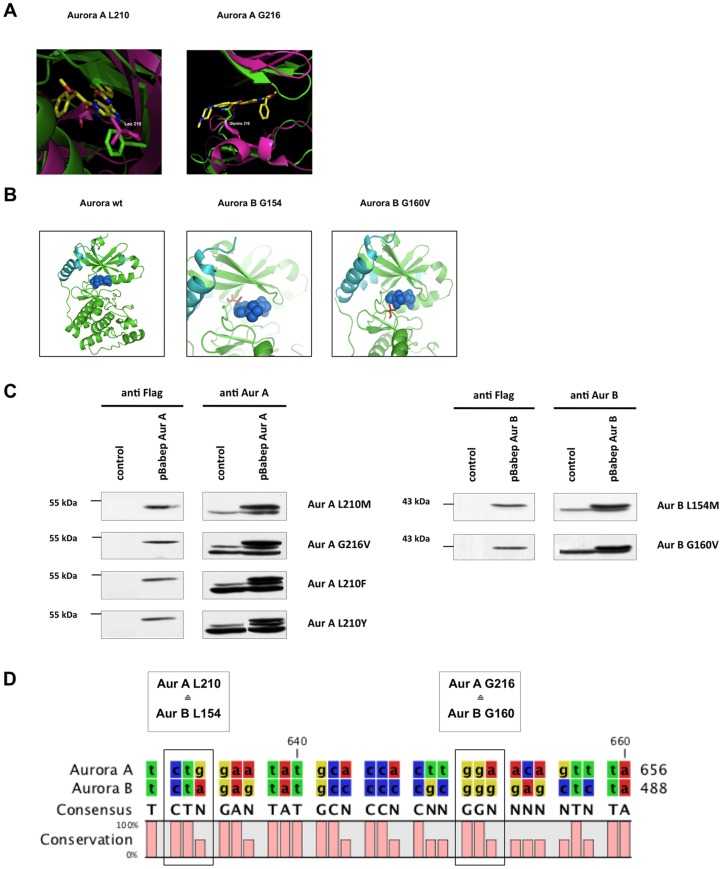
Characterization of Aurora model system. Crystal structure was analyzed in order to identify residues, whose specific mutation should abolish inhibitor binding while keeping Aurora kinase activity. (A) Superimposition of the CK2 crystal structure (green) with Aurora A (pink) - PHA-739358 (yellow) complex crystal structure, showing the position of the point mutation L210 and G216. (B) *Xenpus laevis* Aurora B (green) and INCENP (turquoise) in complex with AS7035369, showing the position of the point mutation L154 and G160. (C) Ba/F3 p185 wt cells were transfected with Babe-puro based retrovirus encoding AurA L210M, AurA G216V, AurB L154M, or AurB G160V point mutations. Empty pBabe-vector was used as control. Selection was accomplished with puromycin. Western blot analysis showing flag-tagged Aurora A and B expression similar to endogenous Aurora A/B protein levels. (D) Amino acid sequence alignment of human Aurora kinase A and B. Mutated residues are framed. It is of note that the point mutations L210M and G216V in Aurora A accord with L154M and G160V in Aurora B.

Our previous results hint to Aurora B as a relevant in-vitro target of PHA-739358 and R763/AS703569. To confirm this assumption, we generated point mutations in Aurora A and B with diminished inhibitor binding, while Aurora kinase activity was not affected. Therefore, we performed in-silico modeling of the Inhibitor/Aurora kinase-complex (Figure 5A, B). This analysis revealed two different positions in Aurora A and B (Figure 5D): L210 in Aurora A, corresponding to L154 in Aurora B, constitutes the gate keeper residue in a small hydrophobic pocket at the back of the ATP-binding side, that can be utilized by small molecule ATP competitors but not by ATP itself [90]. Studies from other protein kinases, where the corresponding gatekeeper residues were mutated to bulkier residues resulted in abolished inhibitor binding [91]–[93]. G216 in Aurora A (G160 in Aurora B) maps to the bottom of the kinase hinge loop. Mutation of this residue into a bulkier amino acid was therefore expected to create direct steric disablement to inhibitor binding without interfering ATP binding. To follow our assumptions we introduced these mutations in Aurora A (L210M, G216V) or Aurora B (L154M, G160V) by site-directed mutagenesis and stably expressed them by transfecting Ba/F3 p185wt cells with pBabe-puro based retroviruses encoding one of these constructs. Recombinant Flag-tagged Aurora A and B constructs were expressed at equivalent levels, similar to the endogenous proteins (Figure 5C).

### Aurora B G160V mutant confers resistance to both inhibitors and partially rescues Aurora B associated cell cycle functions

**Figure 6:**
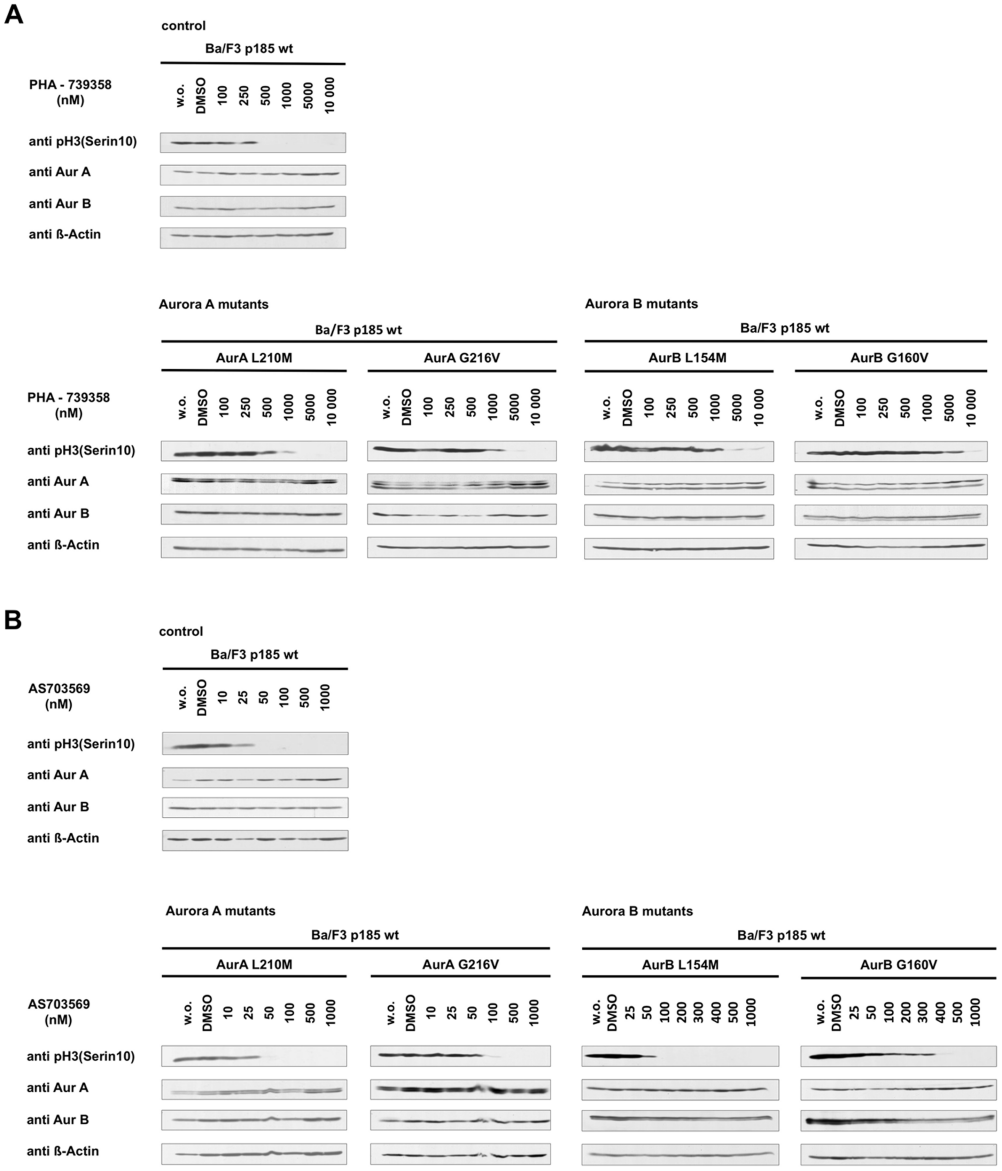
Aurora B G160V mutant confers resistance to PHA-739358 and R763/AS703569. Western blot analysis probed to investigate the resistant potential of Aurora A and B kinase mutants by detecting phospho-Histone-3 (serine 10) after 2.5 h exposure to increasing concentrations of PHA-739358 (A) or AS703569 (B). Untreated and DMSO treated cells were used as control. For comparison untransfected Ba/F3 p185 wt cells were also included into the experiment.

To investigate AKI resistance of the newly engineered Aurora kinase mutants, phosphorylation of Histone-3 was examined after exposure to PHA-739358 or R763/AS703569 (Figure 6 A, B). Interestingly, only AurB G160V expressing cells showed a remarkable resistance to both compounds. Expression of AurA L210M, AurA G216V, AurB L154M and several others Aurora mutations had no or marginal effects (Figure S2). In addition, co-expression of Aurora constructs did not affect BCR-ABL kinase inhibition (Figure S3).

**Figure 7:**
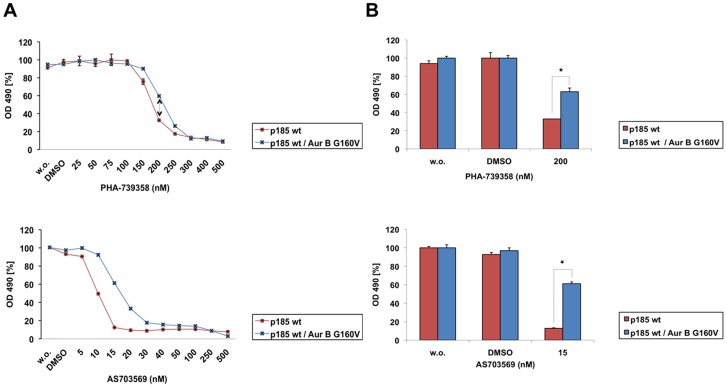
Drug-resistant Aurora B G160V mutant partly overcomes the anti-proliferative effect of PHA-739358 and R763/AS703569. (A) MTT assay showing the anti-proliferative effect after treatment with increasing concentrations of PHA-739358 (upper panel) or R763/AS703569 (lower panel) for 48 h. Percentage of cell growth was normalized to the growth of untreated control cells. Shown is one representative experiment of three experiments performed. (B) Bar graph quantifying cell proliferation after exposure to 200 nM PHA-739358 (upper panel) or 15 nM R763/AS703569 (lower panel) for 48 h. Untreated and DMSO-treated cells were used as control. Shown is one representative experiment of three experiments performed. Values show mean of triplicates ± SD. Difference between Ba/F3 p185 wt and AurB G160V expressing Ba/F3 p185 wt cells is statistically significant.

**Figure 8:**
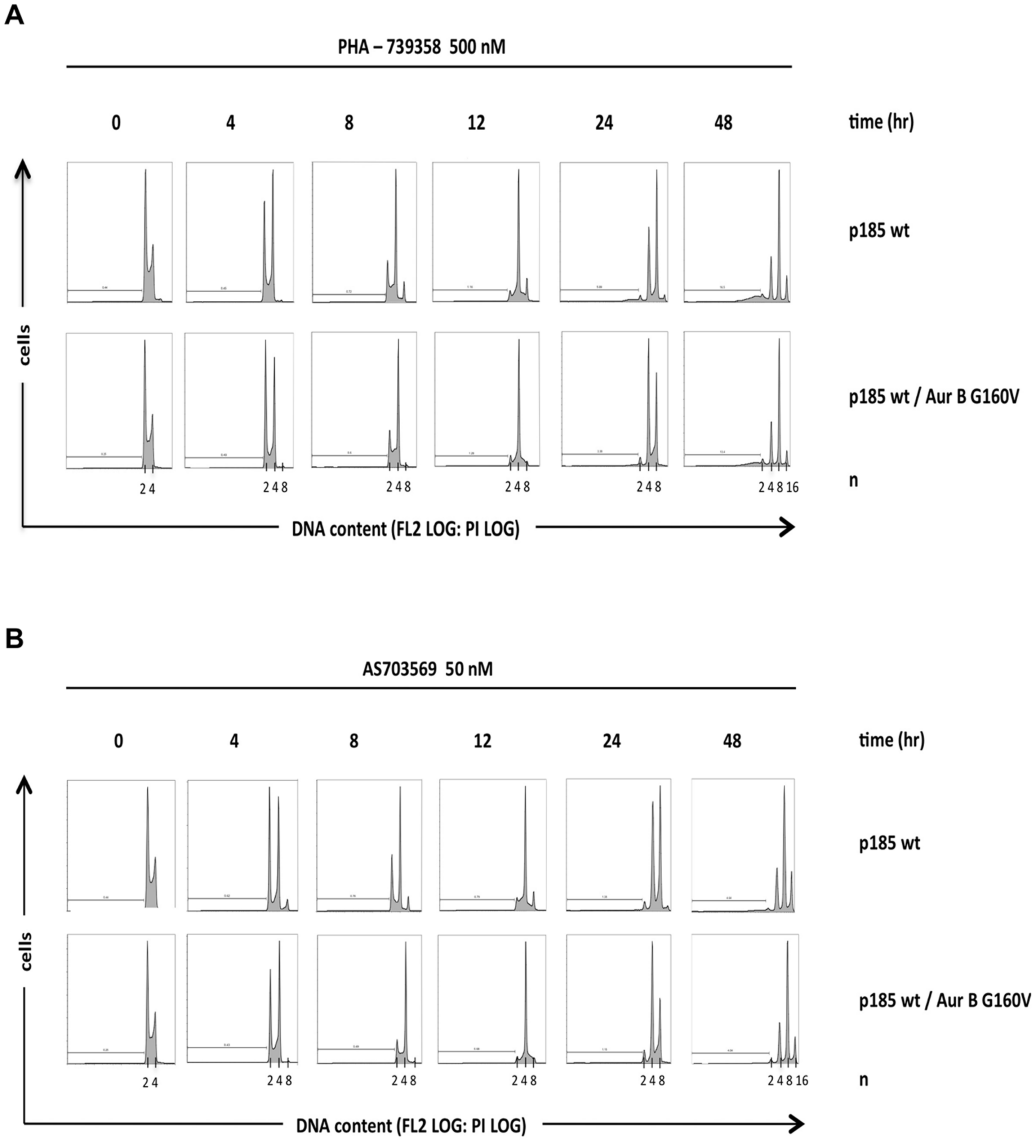
Expression of the resistant Aurora B G160V mutant partly rescues cell division failures after exposure to PHA-739358 and R763/AS703569. Ba/F3 p185 wt and Aur B G160V expressing Ba/F3 p185 wt cells were cultured in the presence of 500 nM PHA-739358 (A) or 50 nM R763/AS703569 (B), harvested at the indicated time points and analysed by flow cytometry to determine DNA content of propodium iodide (PI) stained cells.

To assess whether expression of the drug-resistant AurB G160V mutant reverted in-vitro effects of PHA-739358 and R763/AS703569, we performed proliferation assays and cell cycle analyses. Indeed, AurB 160V attenuated responses to PHA-739358 and R763/AS703569 (Figure 7A, B) and increased IC_50_ values for both drugs in p185 wt cells. Interestingly, cell cycle analysis revealed polyploidisation up to 16n in the presence of PHA-739358 or R763/AS703569 not only in control cells but also in G160V expressing cells. Nevertheless, distinct polyploidisation also became apparent with delay in the AurB G160V mutant: a considerable 8n population was observed after 24 h exposure to PHA-739358 or AS70356 in AurB G160V cells, whereas it was already recognized 8 h after PHA-739358 and 4 h after AS70356 treatment in p185wt cells (Figure 8 A, B). The apoptotic sub-G1 fraction increased over time, with the highest value in control cells 48 h after PHA-739358 treatment.

**Figure 9:**
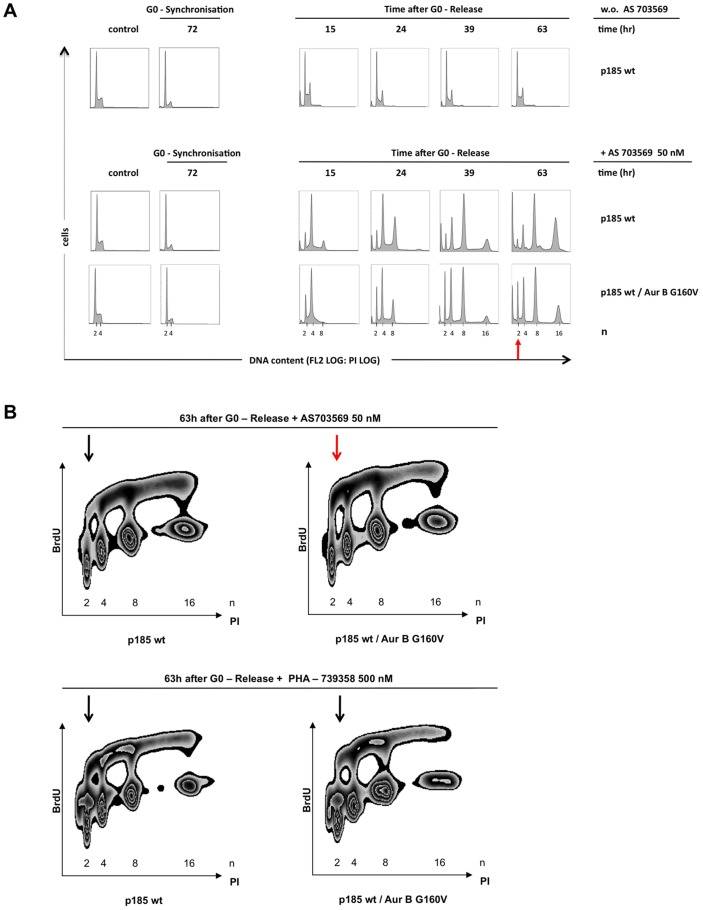
Aurora B G160V mutant constrains endoreduplication and rescues partly a normal cell cycle. Asynchronously growing Ba/F3 p185 wt cells and Aurora B G160V expressing Ba/F3 p185 wt cells (AS) were serum starved for 72 h (SS), then reactivated with 20% serum and simultaneously treated with 500 nM PHA-739358 or 50 nM R763/AS703569 for 63 h. (A) Time course for DNA synthesis after G_0_-release and exposure to 50 nM R763/AS703569. (B) DNA synthesis was monitored by BRDU pulse for 1 h after 63 h of culture. Only in the presence of 50 nM R763/AS703569 (upper panel) and not of 500 nM PHA-739358 (lower pannel) Aurora B G160V mutant restores the 2n population (arrow), demonstrating regeneration of Aurora B kinase activity.

To determine whether the observed effects were a consequence of Aurora B overexpression or resulted from resistance due to the AurB G160V mutant, dynamic 2-dimensional cell cycle analyses were performed. To this end, indicated cell lines were first synchronized in G_0_ by serum deprivation for 72 h and then reactivated by addition of 20% FCS in the presence of PHA-739358 or R763/AS703569 for indicated timepoints. Treatment with PHA-739358 or R763/AS703569 for 63 h resulted in a distinct G_1_-accumulation (2n peak) in AurB G160V expressing cells but not in the control cells, indicating normal cell cycle function in cells harboring the AurB G160V mutation (Figure 9A). Next, dynamic cell cycle experiments were performed, and DNA synthesis was monitored by a BrdU pulse after 62 h for 1 h. In control cells, R763/AS703569 reduced the proportion of cells in S-Phase reduplicating their 2n DNA-content in favor of cells reduplicating their 4n or 8n DNA-content (Figure 9B, left upper panel). Hence, cells exited mitosis without dividing, returned to G_1_ with a 4n DNA-content, kept on DNA-reduplicating and finally lost proliferative potential. Despite polyploidisation, expression of the AurB G160V mutation restored the proportion of cells in S-Phase reduplicating their 2n DNA-content, constrained endoreduplication and consequently partially rescued a normal cell cycle, demonstrating regeneration of Aurora B Kinase activity (Figure 9B, right upper panel). Surprisingly, PHA-739358 treatment of AurB G160V cells was not able to partially override polyploidisation and endoreduplication (Figure 9B, lower panels).

## Discussion

Imatinib has become the gold standard in the treatment of CML with excellent and durable responses and minimal side effects [9], [10], [27], [94]. However, around 25% of patients treated with Imatinib do not respond to treatment or relapse after initial response to Imatinib [95], [96]. 2^nd^ generation ABL-kinase inhibitors such as Nilotinib, Dasatinib, and Bosutinib proved to be effective against a variety of Imatinib-resistant BCR-ABL mutations, but are ineffective against the BCR-ABL T315I mutation [97]–[99]. Since PHA-739358 and R763/AS703569 are active against Aurora kinases as well as BCR-ABL [84], [85], [100]–[102], we intended to dissect the significance of aurora kinase versus BCR-ABL inhibition for anti-proliferative and pro-apoptotic activity of AKIs in BCR-ABL transformed cells harboring known BCR-ABL resistance mutations as well as mutations in Aurora B. It has already been shown, that AKIs like VX-680 are active in patients with BCR-ABL T315I leukemia [103], [104]. Within our study, we could show that Aurora kinases are a valuable potential therapeutic target for kinase inhibitor resistant CML. Both AKIs used in this study display a significant, dose-dependent inhibition of proliferation and induction of apoptosis in BCR-ABL-negative and -positive Ba/F3-cells, including those expressing clinically highly relevant BCR-ABL mutations T315I, Y253F, and F317L. The BCR-ABL mutational status did not affect the anti-proliferative activity of both compounds, and sensitivity to PHA-739358 and R763/AS703569 did not correlate with the degree of imatinib resistance. Both agents have already shown clinical activity in phase-1 trials in patients with advanced solid tumors and hematological malignancies, including patients with CML or Ph^+^ALL, resistant to ABL kinase inhibitor treatment and patients harboring the T315I mutation [102], [105]–[109]. Crystal structure analysis of ABL T315I kinase domain in complex with PHA-739358 provided a possible structural explanation for its activity against ABL T315I. While mutation of threonine to the bulkier AA isoleucine caused a steric hindrance and therewith prevented binding of imatinib, PHA-739358 bound the active confirmation of the kinase domain in the ATP-binding pocket and lacked steric hindrance imposed by this substitution [100], [110]. Interestingly, kinase assays with PHA-739358 showed even a higher affinity for the T315I mutation [100]. In our studies, we found no compelling difference in activity of PHA-739358 or R763/AS703569 in Ba/F3 cells expressing BCR-ABL wild-type or T315I: both inhibitors exerted anti-proliferative and pro-apoptotic activity in all cell lines tested, but at different concentrations. Previous studies indicated nanomolar activity of the used AKIs in this study in preclinical models of solid tumors and hematologic malignancies including BCR-ABL transformed cell lines [101], [111]–[114]. In our experiments, cellular IC_50_- values of PHA-739358 were in the range of 150 nM, whereas IC_50_- values of R763/AS703569 is 15-times lower concentrations in BCR-ABL transformed cell lines. In addition, biologic activities of R763/AS-703569 occur at similar concentrations in the context of different transforming oncogenes (i.e. Myc, BCR-ABL etc. [111] and this work), indicating that Aurora kinases are the primary target in this models. Previous studies with VX-680 already showed [60], [115], that BCR-ABL inhibitory VX-680 concentrations do not appear to be necessary to induce anti-CML activity in human Ph^+^ cell lines suggesting that the activity of VX-680 in CML is mediated at least partly by Aurora- and not by ABL- inhibition. In accordance, we observed anti-proliferative activity of R763/AS703569 at concentrations where inhibition of Aurora kinases but not BCR-ABL kinase occurred. Suppression of phospho-H3, a bone-fide Aurora B target, required R763/AS703569 IC_50_-concentrations, whereas BCR-ABL-inhibition was observed at much higher concentrations. The sufficiency of exclusive Aurora kinase inhibition was confirmed by cell cycle analysis, where treatment with lower, BCR-ABL not inhibiting R763/AS703569 concentrations, resulted in endoreduplication and polyploid cell (>4n) accumulation as a consequence of continued cell cycle progression in the absence of cell division. In vitro experiments with PHA-739358 revealed equivalent biologic results, although higher concentrations of PHA-739358 were used, that inhibit Aurora kinases as well as BCR-ABL. However, identical cell cycle aberrations could be detected by treatment of BCR-ABL-negative cells with both AKIs arguing for an exclusive Aurora kinase-mediated and BCR-ABL-inhibition independent effect. These data corroborate the hypothesis that cytotoxicity of PHA-739358 and AS-703569 primarily depends on inhibition of Aurora kinases associated in-vitro effects.

Both Aurora kinases A and B have been linked to tumorigenesis and previous publication highlight the importance of Aurora A inhibition: Kelly et al. showed preclinical activity of Alisertib, a selective Aurora A inhibitor as a single agent or in combination with nilotinib [76] in CML cells bearing wild-type or T315I BCR-ABL. Moreover, Yuan et al showed that CML resistance can be overcome with specific Aurora A gene knockdown. Next to Aurora A knockdown, this study used the AKI S1451, which spared Histone H3 Ser10 phosphorylation and polyploidy [60], suggesting a specific Aurora A inhibition. It is worth noting, that it could be recently shown, that BCR-ABL induces upregulation of both Aurora kinases A and B via AKT in BCR-ABL positive cells [116], suggesting an impact of both Aurora isoforms in CML cells. Moreover, Mancini et al demonstrated, that VX-680 induced Gadd45a transcription and thereby recruitment of Oct-1 transcription factor at critical promoter regions for gene transcription and covalent modifications of histone H3 (S10 de-phosphorylation, K9 de-methylation and K14 acetylation) [60]. Recent studies have revealed that dephosphorylation of Histone-3 and accumulation of polyploid cells is based on suppression of Aurora kinase B activity [50]–[52], [117]–[120]. Our experiments demonstrated the described Aurora B inhibition phenotype upon PHA-739358 and R763/AS703569 treatment, suggesting that Aurora B may also be a relevant target for both compounds. To confirm this hypothesis, we designed different Aurora B point mutations in the ATP-binding site mediating resistance and were able to identify a PHA-739358 and R763/AS703569 resistant mutant (Aurora B G160V). This mutant shows significant resistance in vitro and in vivo and is able to attenuate the anti-proliferative capacity of both inhibitors in BCR-ABL positive cells, demonstrated by a significant increase of IC_50_- values and a delay in Histone-3 dephosphorylation. Cell cycle analysis detected active Aurora B kinase in the presence of AKIs in Ba/F3 p185 wt Aurora B G160V cells. These findings further substantiate Aurora B kinase inhibition as one cause of the anti-proliferative and pro-apoptotic effects of PHA-739358 and R763/AS703569. Along this line, development of drug resistance arising in cell-cycle kinases is conceivable. Taylor et colleagues [121] reported about acquired point mutations in the Aurora B kinase domain after prolonged exposure to ZM447439. Expression of these mutant Aurora B alleles rendered cancer cell lines resistant to ZM447439 and several other AKIs. However, kinase-independent mechanisms of AKI resistance are also possible. In PHA-739358 resistant cells, overexpression of the Abcg2 efflux carrier could be identified as a potential mechanism of drug resistance [122]. This knowledge should have important implications for future drug-development, focusing on identifying compounds that target mutated Aurora kinases.

Taken together, our data demonstrate that dual ABL and Aurora kinase inhibition can be used to overcome ABL TKI resistant CML. Interestingly, cellular effects of PHA-739358 and R763/AS703569 in BCR-ABL positive cells are primarily mediated by functional inhibition of Aurora kinase B strongly suggesting that Aurora kinases and not BCR-ABL is the biologically relevant target in TKI-resistant Ph^+^ leukemia and that Aurora B may be an essential and attractive target in CML cells.

## Supporting Information

Figure S1 **Expression of Aurora kinase A and B is regulated by BCR-ABL activity.** Ba/F3 p185 wt cells were treated with 2 µM Imatinib for 24 and 48 hours, harvested and determined by western blot analysis with the indicated antibodies. GAPDH served as loading, PY as imatinib control.
(JPG)

Figure S2 **Characterization of further mutants provides no evidence of higher resistance to PHA-739358 or R763/AS703569 than Aurora B G160V mutant.**
(TIFF)

Figure S3 **Expression of Aurora kinase mutations in BCR-ABL positive cells has no influence on the BCR-ABL kinase inhibition concentration of PHA-739358 and R763/AS703569.** Ba/F3 p185 wt cells and the indicated Aurora A and B kinase mutants were treated with increasing concentrations of PHA-739358 (A) or R763/AS703569 (B) for 2.5 h. Phosphorylation levels of BCR-ABL and its downstream target STAT5 were determined by western blot analysis. Untreated and DMSO treated cells served as a control.
(JPG)
