# Supplementary figures and images for: Inhibition of Aurora Kinase B Is Important for Biologic Activity of the Dual Inhibitors of BCR-ABL and Aurora Kinases R763/AS703569 and PHA-739358 in BCR-ABL Transformed Cells
Source: PLoS One. 2014 Nov 26;9(11):e112318. doi: 10.1371/journal.pone.0112318 (PMC4245092; doi:10.1371/journal.pone.0112318)

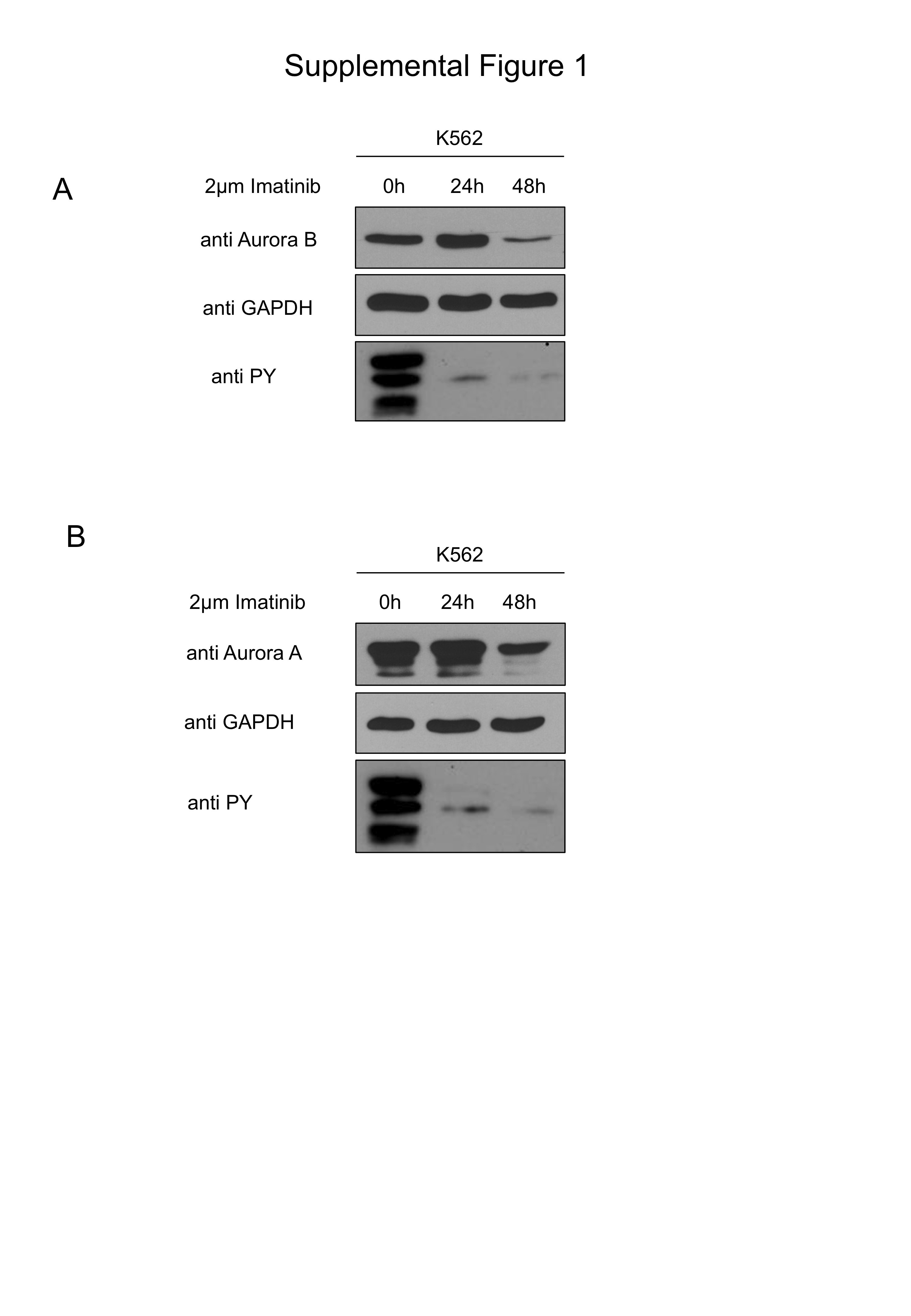

Supplement: Figure S1 — Expression of Aurora kinase A and B is regulated by BCR-ABL activity. Ba/F3 p185 wt cells were treated with 2 µM Imatinib for 24 and 48 hours, harvested and determined by western blot analysis with the indicated antibodies. GAPDH served as loading, PY as imatinib control. (JPG) [file pone.0112318.s001.jpg]

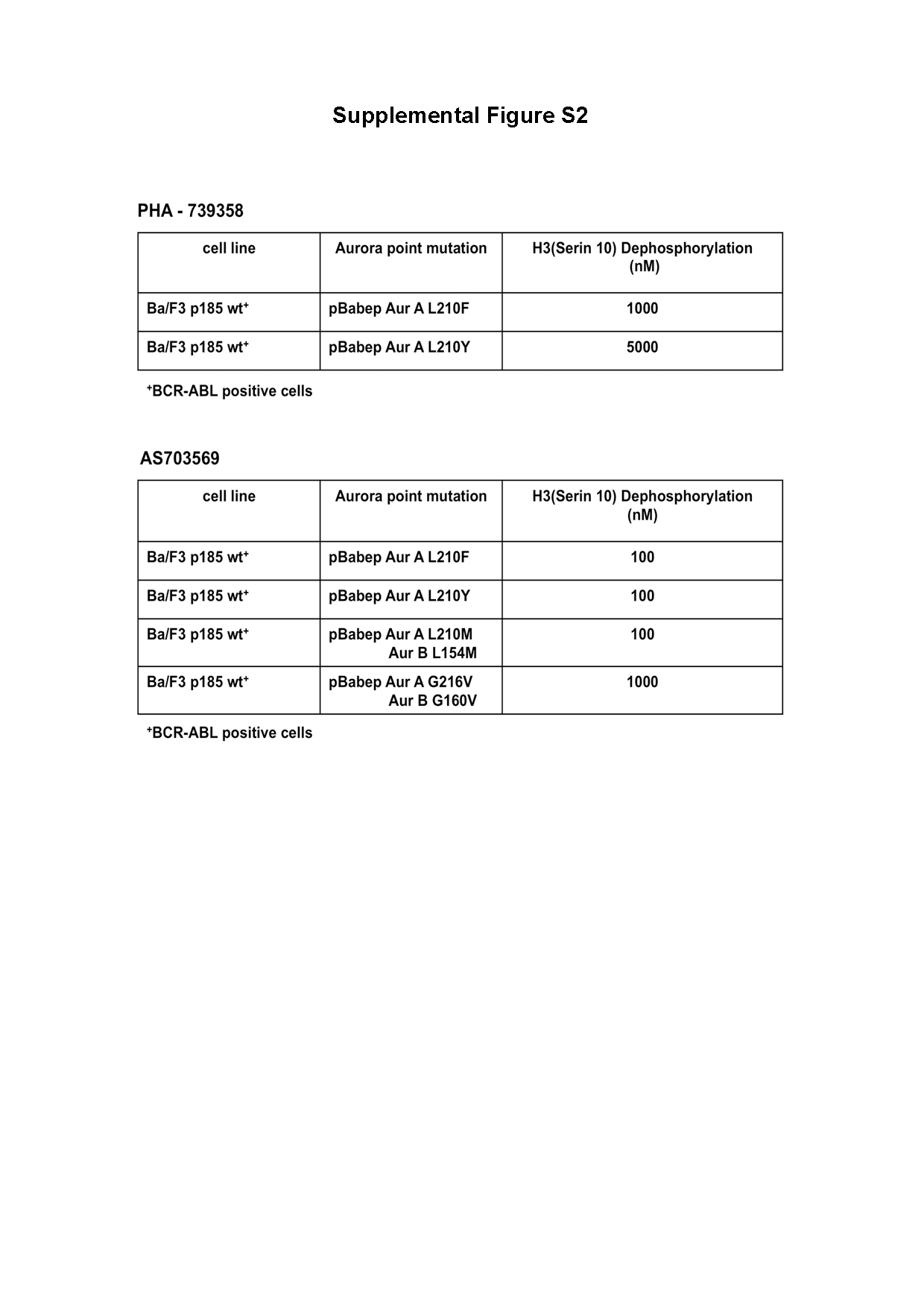

Supplement: Figure S2 — Characterization of further mutants provides no evidence of higher resistance to PHA-739358 or R763/AS703569 than Aurora B G160V mutant. (TIFF) [file pone.0112318.s002.tiff]

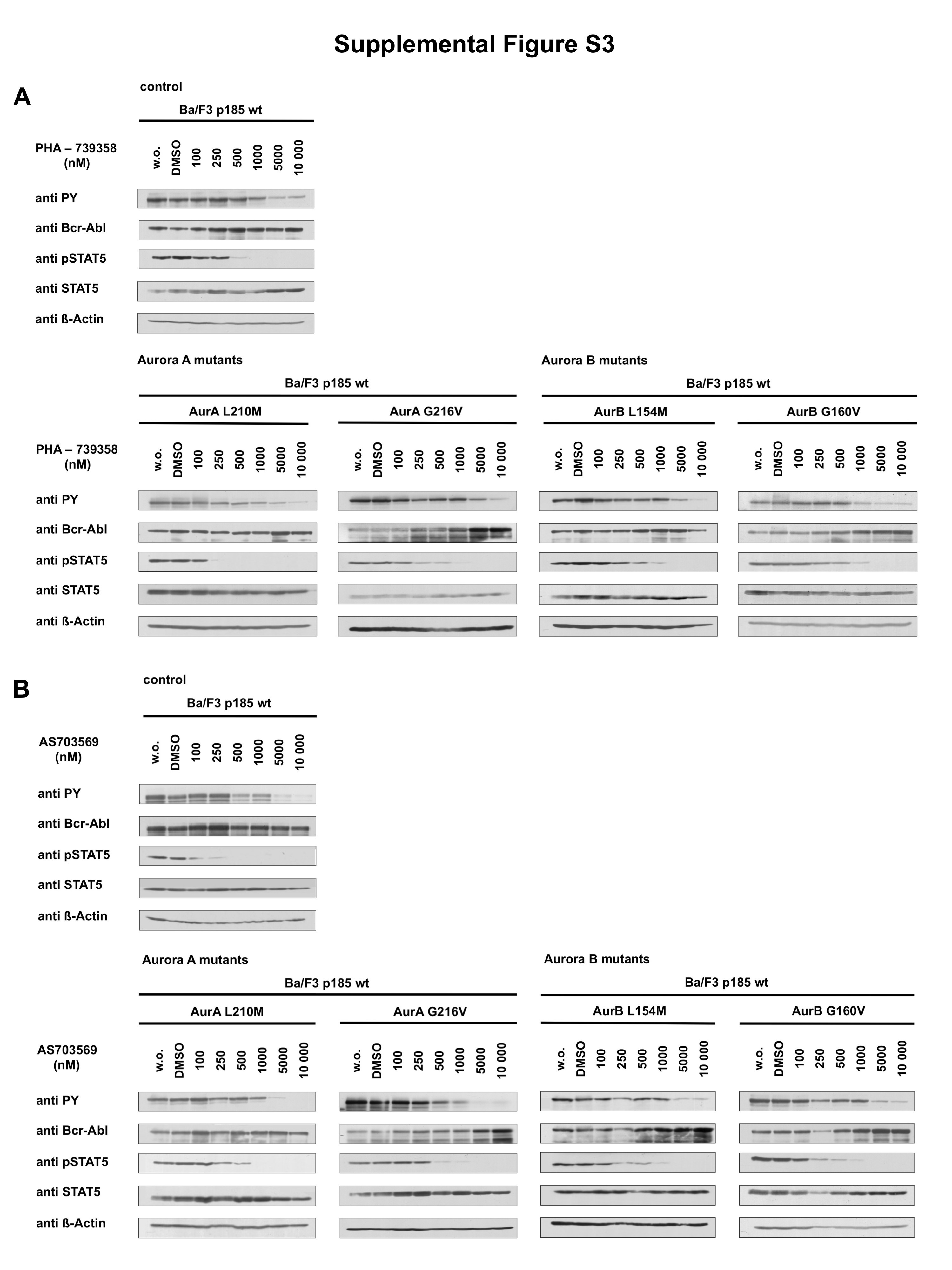

Supplement: Figure S3 — Expression of Aurora kinase mutations in BCR-ABL positive cells has no influence on the BCR-ABL kinase inhibition concentration of PHA-739358 and R763/AS703569. Ba/F3 p185 wt cells and the indicated Aurora A and B kinase mutants were treated with increasing concentrations of PHA-739358 (A) or R763/AS703569 (B) for 2.5 h. Phosphorylation levels of BCR-ABL and its downstream target STAT5 were determined by western blot analysis. Untreated and DMSO treated cells served as a control. (JPG) [file pone.0112318.s003.jpg]
